# Cephalosporin Resistance in *Neisseria gonorrhoeae*

**DOI:** 10.4103/0974-777X.68537

**Published:** 2010

**Authors:** Manju Bala, Seema Sood

**Affiliations:** *Regional STD Teaching Training and Research Centre, Vardhman Mahavir Medical College and Safdarjang Hospital, New Delhi, India*; 1 *Department of Microbiology, All India Institute of Medical Sciences, New Delhi, India*

**Keywords:** Antimicrobial resistance, Cephalosporin resistance, Gonorrhea management, *Neisseria gonorrhoeae*

## Abstract

Gonorrhea, a disease of public health importance, not only leads to high incidence of acute infections and complications but also plays a major role in facilitating human immunodeficiency virus (HIV) acquisition and transmission. One of the major public health needs for gonorrhea control is appropriate, effective treatment. However, treatment options for gonorrhea are diminishing as *Neisseria gonorrhoeae* have developed resistance to several antimicrobial drugs such as sulfonamides, penicillin, tetracyclines and quinolones. Antimicrobial resistance (AMR) surveillance of *N. gonorrhoeae* helps establish and maintain the efficacy of standard treatment regimens. AMR surveillance should be continuous to reveal the emergence of new resistant strains, monitor the changing patterns of resistance, and be able to update treatment recommendations so as to assist in disease control. Current treatment guidelines recommend the use of single dose injectable or oral cephalosporins. The emergence and spread of cephalosporin resistant and multi drug resistant *N. gonorrhoeae* strains, represents a worrying trend that requires monitoring and investigation. Routine clinical laboratories need to be vigilant for the detection of such strains such that strategies for control and prevention could be reviewed and revised from time to time. It will be important to elucidate the genetic mechanisms responsible for decreased susceptibility and future resistance. There is also an urgent need for research of safe, alternative anti-gonococcal compounds that can be administered orally and have effective potency, allowing high therapeutic efficacy (greater than 95.0% cure rate).

## INTRODUCTION

Sexually transmitted infections (STIs), both ulcerative and non ulcerative, constitute one of the major public health problems. There has been increasing world wide incidence of these infections because of several socioeconomic factors like women’s emancipation, permissiveness, homosexuality, population migration and technical factors like increased availability of better diagnostic facilities. The World Health Organization (WHO) estimates that approximately 340 million new cases of the four main curable STIs (gonorrhea, chlamydial infection, syphilis and trichomoniasis) occur every year, majority of them in developing countries.[1]

Gonorrhea caused by *Neisseria gonorrhoeae (N. gonorrhoeae)*, is one of the most common STIs and is a global health problem.[2] Gonorrhea is an easily curable STI. However, undetected, untreated infections can lead to complications like pelvic inflammatory disease, ectopic pregnancy, tubal factor infertility, adverse pregnancy outcomes in females, and testicular and prostate infections and infertility in males. Also, asymptomatic patients, unaware of their infection, may serve as a reservoir of infection to their partners.

Gonorrhea has gained tremendous importance in the last few decades because of its role as a co-factor in increasing HIV infections. This is thought to result from an increase in the viral load in the semen or cervico-vaginal fluids from those co-infected with gonorrhea and HIV, and to an increase in the number of target cells for HIV in the inflammatory exudates present in symptomatic bacterial sexually transmitted diseases (STDs).[3–6] This new association provides an important reason for proper and timely treatment of gonorrhea.

The incidence/prevalence rates of gonorrhea are difficult to ascertain because resources are mostly lacking where the disease is concentrated.[2] Some available estimates of incidence suggest that approximately 62 million new cases of gonorrhea occur globally each year.[2] Despite a high prevalence of gonorrhea, no regular monitoring of antimicrobial susceptibility of *N. gonorrhoeae* is carried out in many countries, the reasons being invasive specimen collection procedures, fastidious nature of the organism, need for specialized culture media and trained personnel.[7] Moreover, as a consequence of emphasis on syndromic management of STDs and the introduction of nonculture-based diagnostic tests, there is decreased availability of *N. gonorrhoeae* isolates for susceptibility testing.

A major contributing factor to the continued spread of gonococcal infections is the remarkable ability of *N. gonorrhoeae* to acquire resistance to antibiotics. Over the last two decades, *N. gonorrhoeae* strains have developed high level of resistance against several antimicrobial agents like penicillin, tetracycline and quinolones in different countries,[8–13] The emergence of strains resistant to extended-spectrum cephalosporins, the antibiotics used as the first line treatment for uncomplicated gonococcal infections, is now a serious concern worldwide as it may pose a problem in the management of gonorrhea. This review is aimed at analyzing the problem of antimicrobial resistance in *N. gonorrhoeae*, particularly to currently recommended cephalosporins.

## MATERIALS AND METHODS

A Medline search was conducted in July (2009), using PubMed, for articles published under the major headings of “antimicrobial resistance in *N. gonorrhoeae*”, “Surveillance of antimicrobial/drug resistance in *N. gonorrhoeae*” and “gonorrhea treatment/therapy,”. Additional searches were performed for related reports posted on the internet by internationally recognized public health agencies. The articles identified through the methods described above were compiled.

### Magnitude of antimicrobial resistance in *N. gonorrhoeae*

Many antimicrobials were active against *N. gonorrhoeae*. However, with the emergence of antimicrobial resistance only few antibiotics are effective against *N. gonorrhoeae*. Single dose therapy is the universal practice of choice in the treatment of uncomplicated gonorrhea. WHO and Center for Disease Control and Prevention (CDC) recommend a change in the treatment regimen when the prevalence of antimicrobial resistance exceeds five per cent for a specific antibiotic.[14,15]

Sulfonamides were used for gonococcal treatment after their introduction in 1936, but their efficacy was short-lived because of the rapid emergence of resistance by 1945[16,17] Penicillin proved highly effective in treating gonorrhea when introduced in the early 1940s. With the emergence and spread of penicillinase producing *N. gonorrhoeae* (PPNG), in the 1970s, a switch to alternative therapy became a necessity in many locations in the 1980s.[18–21] Chromosomally mediated resistance *N. gonorrhoeae* (CMRNG) and PPNG still pose a major problem.

Tetracyclines, being cheap, were widely used in some developing countries. Requirement of a multi-dose regime and compliance with multi-day regimens was usually unsatisfactory.[22,23] Tetracycline resistant *N. gonorrhoeae* (TRNG) were first reported in the United States in 1985.[24] Tetracycline resistance, both chromosomal and plasmid mediated, increased and has attained a high prevalence in many countries such as United States, Trinidad and Tobago, India, Thailand and Indonesia.[25–29] Tetracyclines remain important and effective agents in the treatment of other sexually transmitted infections, notably *Chlamydia trachomatis*.

Fluoroquinolones became popular therapy during the 1980s and were widely used as effective oral therapy against penicillin-resistant *N gonorrhoeae*. The most widely used quinolone against uncomplicated gonorrhea was ciprofloxacin and the efficacy of this agent was 100% with single dose of 500 mg. On the basis of data regarding high efficacy, safety, and convenience as single-dose therapies, oral fluoroquinolones were recommended for gonorrhea treatment by CDC in 1993.[30] Other quinolones, such as ofloxacin and fieroxacin, also showed good clinical efficacy.[31,32] Quinolones were effective in eradicating rectal and pharyngeal infection.[33] Resistance to fluoroquinolones was first detected in mid-1980s in Asia, and then it spread to other areas in Europe, Western Pacific, South and South-East Asia, and the Americas.[9–11,34–37]

## CURRENT TREATMENT GUIDELINES FOR GONORRHEA

Presently, the recommended first-line treatment for gonorrhea in most countries includes antibiotics such as cefixime, ceftriaxone and in some cases spectinomycin, azithromycin.

### Cephalosporins

Cephalosporins, in the form of “third-generation” preparations, have proved highly effective for more than a decade in the treatment of gonorrhea, including PPNG and chromosomally-mediated penicillin resistance and are currently recommended for treatment of gonorrhea. Ceftriaxone is recommended as the drug of choice for gonorrhea as it is safe and effective for the treatment of uncomplicated gonorrhea at all anatomical sites. It has been reported to cure 98.8% of uncomplicated urogenital and anorectal infections in published clinical trials.[38] Ceftriaxone is favored in comparison with other cephalosporins for its long serum half life, and side-effects are infrequent and generally mild. The dose recommended by CDC and WHO is 125 mg intramuscularly (IM).[14,15] However, many countries recommend 250 mg and in China and Japan, the dose recommended is one gram ceftriaxone.[39–41] Cefotaxime 500 g IM as a single dose is an alternative preparation of proven efficacy.[30] These cephalosporins regimens have shown good efficacy against rectal and pharyngeal infection.[42] The drawbacks of these highly effective regimens include expense, the necessity to administer them by injection and discomfort at the injection site.

Cefixime is an oral preparation with similar spectrum to that of ceftriaxone. A single oral dose of cefixime 400 mg has been shown to be of equivalent efficacy to ceftriaxone.[43] There is more extensive use of injectable ceftriaxone in comparison to oral cefixime.[41]

Other injectable and oral cephalosporins are available but do not offer any substantial advantages over ceftriaxone and cefixime. Where these specific antimicrobials are not available, a variety of other cephalosporins have proven efficacy in the treatment of urogenital and anorectal gonorrhea. Possible injectable alternatives include cefotaxime, ceftizoxime, and cefodizime. Oral alternatives to cefixime are ceftibuten, cefdinir, cefpodoxime proxetil, cefoperozone, cefditoren and cefuroxime axetil. The pharmokinetics of cefuroxime axetil (one gram oral) are suboptimal as a single dose treatment.[44] Although clinical trial data on cefpodoxime (400mg oral) was very limited in earlier years,[45] cefixime was not available in the USA from 2002 to 2008 and cefpodoxime 400 mg was more widely used during that time.[46] Oral ceftibuten is being used In Hong Kong since 1997 and cefditoren and cefdinir in Japan.[47,48]

### Spectinomycin

Spectinomycin played a central role in the control of gonococcal infection following the emergence of PPNG and higher-level chromosomal resistance. Adoption of spectinomycin as the routinely used drug of choice was soon followed by reports of spectinomycin resistance.[49] Spectinomycin resistance is unstable and reverts once its use is discontinued. It is highly effective as a single intramuscular dose of two grams for urethral and cervical Infection. It has poor efficacy against pharyngeal infection.[33] It remains a useful reserve option for gonococcal therapy and is generally reserved for situations where cheaper alternatives are contraindicated, for example in a pregnant woman who is allergic to cephalosporins.[50] In Japan, where oral cephalosporin resistance is common, it has been shown to be effective for treatment of gonococcal infections.[51] It is, however, expensive and not available in some countries like India.[52] There have been recent reports of occasional spectinomycin resistant isolates from some countries like US, India, WHO Western Pacific region and China.[53–56]

### Azithromycin

Azithromycin is a newer antibiotic belonging to a class of compounds known as azalides, which resemble macrolides. It achieves a high, prolonged, intracellular concentration and has potential as an alternative, effective, oral therapy for gonorrhea. It is effective in single dose therapy against genital infection with *C. trachomatis*.[57,58] It is active *in vitro* against *N. gonorrhoeae* and recent studies show promising efficacy *in vivo* using a single oral dose. Single-dose treatment with two grams gave a 98-99% efficacy in uncomplicated gonococcal infection, whereas smaller studies using a single dose of one gram showed marginally lower efficacy.[59,60] CDC does not recommend one gram dose, as it can cause rapid emergence of antimicrobial resistance.[61–65] The two gram dose was associated with a high frequency (35%) of gastrointestinal side effects, which were generally mild.[59]

Azithromycin proved highly effective (100%) against pharyngeal infection and penicillin-resistant strains.[59] All co-infections with *C. trachomatis* were cured. Resistance to two-gram azithromycin is increasing with high-level resistance recently reported in the US, UK and Scotland.[66–68]

### Emergence of cephalosporin resistance in *N. gonorrhoeae*

Clinical failure with oral cephalosporins was first documented in Japan in two patients with gonococcal urethritis.[69] Subsequently, resistance to oral third generation cephalosporins was reported from many areas in Japan.[70,71] Therefore, in Japan, cefixime was discontinued as a drug of choice for gonorrhea in 2006.[72] Ceftriaxone and spectinomycin, both injectable, are recommended as first-line therapy for gonorrhea in Japan.

Decrease in susceptibility to cephalosporins was also noted in many countries like India, US, other countries in the WHO Western Pacific Region (Australia, Brunei, China, and Papua New Guinea), Vietnam and Greece.[12,73–77] Resistance to oral cephalosporins have been reported from Hong Kong and Taiwan.[47,78] In Hong Kong, clinical treatment failure rate of empirical ceftibuten was around 3.7%, which is still within the five per cent figure generally considered an acceptable resistance level.[47] All the isolates in this study remained susceptible to ceftriaxone by laboratory testing criteria. So far, no treatment failures with injectable ceftriaxone have been reported.[12,47,79] Gonococci may be clinically resistant to orally administered extended spectrum cephaosporins (ECS) while remaining sensitive to the injectable ceftriaxone. It was proposed that the two groups of ESC should be considered separate treatment entities for definitional purposes.[79]

Some of these reports have documented that these ceftrixoneless susceptible strains were multi drug resistant.[12,73,77,80–82] All these strains were susceptible to spectinomycin.

### Problem of multi drug resistant *N. gonorrhoeae*

Many studies have reported multi drug resistant *N. gonorrhoeae* (MDR-NG).[12,73,77,80–83] However, recently, there have been concerns for defining MDR-NG as the term MDR is being used without precise definition.[73,79] In earlier definitions of MDR, resistance to out-dated or little-used drugs like tetracycline was included and multiresistant isolates were defined as quinolone resistant *N. gonorrhoeae* (QRNG) and PPNG; QRNG and TRNG; QRNG, PPNG and TRNG; QRNG and azithro resistant.[12,83] MDR-NG by the revised criteria, are defined as those resistant to one of the antibiotic classes listed in category I (injectable cephalosporins/oral cephalosporins/spectinomycin), plus two or more in category II (Penicillins/ Fluoroquinolones/Azithromycin/Aminoglycosides/Carbapenems).[80] Extensively-drug resistant *N. gonorrhoeae* (XDR-NG) include those resistant to two or more of the antibiotic classes in category I and three or more in category II.[80] XDR-NG are yet to be reported.[80] If the current guidelines for treatment of gonorrhea and concomitant control of gonococcal disease i.e. the usage of right drugs at the right time in the right dose are not adhered to, will definitely lead to the spread of existing MDR-NG and, presumably, the emergence of XDR-NG.

### Mechanism of resistance to cephalosporins and other antimicrobials

Many studies of molecular mechanisms that underlie resistance to various classes of antimicrobial agents have been reported.[84] Mutations in the *gyrA* and *parC* genes are responsible for resistance to fluoroquinolones in *N. gonorrhoeae*.[85,86] In addition, alterations in drug permeation and drug efflux can contribute to the level of resistance to fluoroquinolones.[87] The latter mechanisms are associated with the development of cross-resistance to structurally unrelated antibiotics.[87] Therefore, the resistance to azithromycin has been linked to the multiple transferable resistance (mtr) efflux system.[88,89]

Resistance to spectinomycin can be the result of a single step mutation, possibly due to mutations in the 16S rRNA gene.[90] The mechanisms for chromosomally mediated resistance to penicillin G and tetracycline in *N. gonorrhoeae* involve the *penA*, *penB*, and *mtr* mutations. Mutations in *penA* causes insertion of a single amino acid into penicillin-binding protein 2 (PBP 2), and this reduces the level of binding of penicillin to PBP 2.[91] The *penB* mutation, which is a mutation that is linked to the porin gene, reduces porin permeability to hydrophilic antibiotics and plays an important role in the development of resistance to penicillin G, cephalosporins, and tetracycline.[92] m*tr* increases the level of expression of the MtrCDE efflux pump and confers resistance to multiple hydrophobic agents (i.e.,crystal violet, Triton X-100, and erythromycin) and some hydrophilic antibiotics such as the penicillins.[93]

Recently, *ponA1* and another resistance locus, termed *penC*, were shown to be involved in penicillin resistance. *ponA1* encodes altered PBP 1 and *penC* mutations now named as *pilQ2* mutations, is required to transform an intermediate-level penicillin-resistant strain with *ponA1* to high level resistance.[94] The reduced susceptibility of *N. gonorrhoeae* strains to broad spectrum cephalosporins such as cefixime and ceftriaxone has been proposed to be associated with polymorphisms in several of these genes and especially with certain *penA* mosaic alleles.[81,95] These mosaic sequences are thought to have evolved from recombination events involving *penA* gene sequences from several *Neisseria* species, including *N. perflava*, *N. sicca*, and *N. cinerea*.[95,96] Therefore, reduced susceptibility to newer cephalosporins is attributed to the acquisition of genetic material from resistant commensal *Neisseria* spp. by originally susceptible gonococci. However, thorough knowledge regarding these molecular mechanisms is still lacking. All these genes need to be systematically sequenced in more numerous and evidently diverse clinical *N. gonorrhoeae* strains with reduced susceptibility to broad-spectrum cephalosporins.[97]

### Future options in treatment of *N. gonorrhoeae* infections

The worldwide increase in resistance of *N. gonorrhoeae* to all classes of antimicrobials is of serious concern and necessitates the search of alternative remedies for the treatment of gonorrhea. There is an urgent need for safe, alternative anti-gonococcal compounds that can be administered orally and have effective potency, allowing high therapeutic efficacy (greater than 95.0% cure rate) with preferably a single-dose regimen. However, very little research regarding this is being carried out. Recently, activities of some medicinal plants have been evaluated against *N. gonorrhoeae* which seems to have a promising future.[98–100] Among the compounds evaluated, eugenol, a compound from *Ocimum sanctum* was also found to be active against multi resistant isolates of *N. gonorrhoeae*.[101]

Two studies evaluated the activities of topical microbicides.[102,103] In one of these studies, a polyherbal cream (Basant) inhibited the growth of WHO strains and clinical isolates of *N. gonorrhoeae*, including those resistant to penicillin, tetracycline and ciprofloxacin.[103]

Research is needed to evaluate the activity of other antimicrobials or combinations of antimicrobials that may be efficacious for the treatment of urogenital and anorectal gonorrhea.

## CONCLUSION

Increase in *N. gonorrhoeae* isolates which are resistant to multiple antimicrobial agents including oral cephalosporins and the emergence of intermediate-level resistance to the injectable ephalosporins is now a serious problem. If this form of resistance evolves further, it will pose a major threat to public health. Therefore, efficient methods for its detection and control will need to be in place. This has led to renewed calls for better control of gonococcal disease, including enhanced global surveillance of resistance.[104,105] Although considerable technical and logistical difficulties are associated with this approach, the WHO has already expanded its regional surveillance programs and consolidated the reporting and analysis of data generated.[80] This also underscores the importance of the cautious use of antibiotics, and there is a need for the development of a wider range of antimicrobial options.
